# First-row transition metal doped germanium clusters Ge_16_M: some remarkable superhalogens†

**DOI:** 10.1039/d1ra08527a

**Published:** 2022-05-04

**Authors:** Huu Tho Nguyen, Ngo Tuan Cuong, Ngo Thi Lan, Nguyen Thanh Tung, Minh Tho Nguyen, Nguyen Minh Tam

**Affiliations:** Faculty of Natural Sciences Education, Sai Gon University 273 An Duong Vuong Street Ho Chi Minh City Vietnam; Center for Computational Science, Faculty of Chemistry, Hanoi National University of Education Hanoi Vietnam; Institute of Materials Science and Graduate University of Science and Technology, Vietnam Academy of Science and Technology 18 Hoang Quoc Viet Hanoi Vietnam; Institute of Science and Technology, TNU-University of Sciences Tan Thinh Ward Thai Nguyen City Vietnam; Institute for Computational Science and Technology (ICST) Quang Trung Software City Ho Chi Minh City Vietnam; Laboratory of Theoretical and Computational Biophysics, Advanced Institute of Materials Science, Ton Duc Thang University Ho Chi Minh City Vietnam nguyenminhtam@tdtu.edu.vn; Faculty of Pharmacy, Ton Duc Thang University Ho Chi Minh City Vietnam

## Abstract

A theoretical study of geometric and electronic structures, stability and magnetic properties of both neutral and anionic Ge_16_M^0/−^ clusters with M being a first-row 3d transition metal atom, is performed using quantum chemical approaches. Both the isoelectronic Ge_16_Sc^−^ anion and neutral Ge_16_Ti that have a perfect Frank–Kasper tetrahedral *T*_d_ shape and an electron shell filled with 68 valence electrons, emerge as magic clusters with an enhanced thermodynamic stability. The latter can be rationalized by the simple Jellium model. Geometric distortions from the Frank–Kasper tetrahedron of Ge_16_M having more or less than 68 valence electrons can be understood by a Jahn–Teller effect. Remarkably, DFT calculations reveal that both neutral Ge_16_Sc and Ge_16_Cu can be considered as superhalogens as their electron affinities (≥3.6 eV) exceed the value of the halogen atoms and even that of icosahedral Al_13_. A detailed view of the magnetic behavior of Ge_16_M^0/−^ clusters shows that the magnetic moments of the atomic metals remain large even when they are quenched upon doping. When M goes from Sc to Zn, the total spin magnetic moment of Ge_16_M^0/−^ increases steadily and reaches the maximum value of 3 *μ*_B_ with M = Mn before decreasing towards the end of the first-row 3d block metals. Furthermore, the IR spectra of some tetrahedral Ge_16_M are also predicted.

## Abstract 2

A theoretical study of geometric and electronic structures, stability and magnetic properties of both neutral and anionic Ge_16_M^0/−^ clusters with M being a first-row 3d transition metal atom, is performed using quantum chemical approaches.

## Graphical abstract

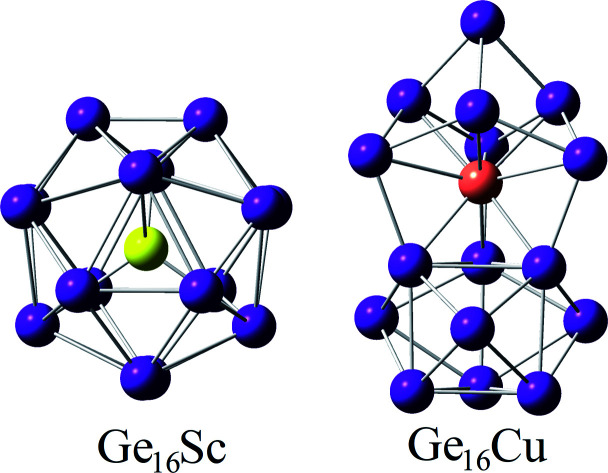

## 1. Introduction

Along with silicon, germanium is one of the most important microelectronic materials. The last several decades have witnessed a continuing interest in the clusters of this semiconductor element since their bulk materials can no longer satisfy the current needs of the miniaturization of electronic devices.^1–11^ Both silicon and germanium do not favor sp^2^-hybridization such as carbon whose small clusters tend to form linear or planar cyclic structures, but rather prefer 3D species arising from a tetrahedral sp^3^ hybridization. Consequently, pure germanium clusters with high symmetry are often unstable in the form of empty caged structures. Since the first observation of the far more abundant Si_15_M and Si_16_M that was reported in 1987 from the laser photoionization time of flight mass spectra,^12^ subsequent investigations using *ab initio* calculations on the geometrical and electronic structures of the Si_15_ and Si_16_ clusters doped with several transition metals such as Cr, Mo, and W have been performed.^13^ These studies showed that the transition metal dopant is often located inside a polyhedral cage forming the Si_15_M and Si_16_M clusters that have high thermodynamic stability and low magnetic moments as compared the M metal dopants. In a recent review article, Kumar *et al.*^14^ analyzed in detail the electronic and geometrical structures of the Si_15_ and Si_16_ clusters doped with several transition metals and showed that the transition metal atom is always endohedrally doped within the silicon cage. This thus demonstrates that introduction of hetero-atoms as dopants into hollow cages can supply us with a valuable pathway to stabilize endohedral cage-like clusters as well as to adjust their many novel physico-chemical properties. Motivated by such a fundamental feature, along with several studies performed on doped silicon clusters, a large number of both experimental and theoretical investigations on germanium clusters doped by various chemical elements have been carried out.^15–39^

Transition metal atoms that have unpaired valence electrons in their *n*d electronic configurations, are inherently magnetic elements. They have been considered as interesting dopants in clusters since interactions between these impurities and the host are expected to alter both electronic and geometrical structures and thereby to generate the doped clusters possessing some novel physico-chemical properties. Moreover, as stated above, due to their high coordination number, transition metals can endohedrally be doped and stabilize the caged structures and simultaneously tailor magnetic properties of host clusters. Indeed, previous studies of singly transition metal doped germanium clusters showed that starting from the size *n* = 9, the Ge unit absorbs the Ni and Ru dopant endohedrally in giving rise to the most stable isomers of Ge_*n*_Ni and Ge_*n*_Ru.^22,24^ The metal atom is encapsulated inside a germanium cage at *n* = 10 when the dopant is Ti, V, and Cu,^20,25,29^ and the critical size for the heavy metal W atom being completely enclosed into a caged germanium framework in the Ge_*n*_W clusters turns out to be at *n* = 12.^33^ A theoretical investigation^34^ on divalent-metal atom doped silicon, germanium and tin clusters X_*n*_M (X = Si, Ge, Sn; *n* = 8–12 and 14) demonstrated that the 12- and 14-atom clusters can be transformed into magic clusters upon doping. Particularly, the manganese-doped X_12_Mn was found to be an icosahedral superatom with a high magnetic moment of 5 *μ*_B_.^34^ In an examination of doubly iron-doped germanium clusters, Liang and co-workers also indicated that both neutral and cationic states of Ge_*n*_Fe_2_^0/−^ adopt polyhedral cage-like shapes with one Fe atom located inside the cage with 9 ≤ *n* ≤ 12.^26^ Soon after the theoretical prediction of metal-encapsulated silicon cages,^40^ Kumar and Kawazoe performed a series of calculations to explore the possible germanium cages stabilized by metal doping. Analogous to M@Si_*n*_ clusters, they explored M@Ge_*n*_ (*n* = 14–16 and M = Ti, Zr, Hf, Fe, Ru, Os) clusters with various possible cage configurations such as the Frank–Kasper (FK) polyhedron, capped decahedron, fullerene-like cage and cubic cage.^41–44^ Remarkably, the Ge_16_M sizes have been one of the most attractive germanium clusters that have been reported so far. A quantum chemical study of Kumar *et al.*^41^ revealed that the very stable ground states of Ge_16_M clusters, with M being elements of Group IVb including Ti, Zr, and Hf, are FK tetrahedra characterized by large HOMO–LUMO energy gaps. Surprisingly, the energy gap for Ge_16_Zr is even larger than the value for the lowest-energy isomer of FK Si_16_Zr discovered before.^40^ Most recently, Du and co-workers carried out an investigation on the interaction in dimers of well-known endohedrally doped clusters, including several X_16_M clusters with X being the tetravalent elements, and found that Ge_16_Ti cage clusters emerge as suitable building blocks to assemble generating solids and nanostructures with enhanced stabilities and diverse physical properties.^45^

To date, many transition metal-silicon and transition metal-germanium clusters have been examined, and the understanding on the cage-like silicon and germanium structures stabilized by doping of some transition metals has well been established on the basis of the concept of filling the electron shells for superatoms within a spherical potential model, and also of various electron counting rules including the Wade–Mingos rules, systems with 18 and 32 electrons.^14^ However, to the best of our knowledge, until recently only one study on the trimeric Ge_2_M including all 3d transition metals M was reported.^46^ Therefore, systematic theoretical studies on a certain series of transition metal-germanium clusters are still necessary in order to understand more deeply the relationship between structures and electronic properties of the transition metal doped Ge cluster, especially the Ge_16_ ones bearing the characteristic Frank–Kasper geometry. In this context, we set out to perform a systematic theoretical investigation on the geometries, stability, and magnetic properties of the germanium clusters doped by one atom belonging to the 3d row transition metals in both neutral and anionic states Ge_16_M^0/−^, with M going from Sc to Zn. Using density functional theory (DFT) calculations, we thoroughly determine the geometries of the lowest-lying equilibrium structures and thereby explore their structural evolution, as well as assign their electronic configurations, energetic parameters and magnetic properties. In particular, some systems behaving as strong superhalogens are discovered.

## 2. Computational methods

On the basis of a reliability test that has been obtained from a previous study on germanium-based clusters,^21^ we select the hybrid B3PW91 functional in conjunction with the 6-311+G(d) basis sets as implemented in Gaussian 09 package^47^ for all electronic structure calculations carried out in this work. The unrestricted formalism is used for species with an open electronic shell. The search for local energy minima is conducted using the two approaches. First, plausible structures of Ge_16_M clusters are generated using a stochastic algorithm,^48^ which was improved based on the random kick procedure reported by Saunders.^49^ By another way, initial structures of each Ge_16_M are manually constructed by adding the M atom at all possible positions on the surfaces of the reported low-lying isomers of Ge_16_.^10,11^ The initial guess structures are then geometrically optimized using the hybrid B3PW91 functional in conjugation with the small LANL2DZ basis set. Several local minima obtained by different approaches turn out to be identical. The local energy minima having relative energies of <5 eV with respect to the lowest-lying isomer are then reoptimized at different spin states using the same B3PW91 functional but with the larger 6-311+G(d) basis set. Harmonic vibrational frequencies and zero-point energy (ZPE) corrections of the Ge_16_M clusters are subsequently calculated at the same level. Unless otherwise stated, relative energies quoted hereafter are determined from B3PW91/6-311+G(d) + ZPE computations.

Furthermore, the natural bond orbital (NBO) analyses are performed by using the NBO 3.0 program implemented in the Gaussian package to examine the electronic configuration and thereby rationalize the magnetic and chemical bonding properties of the clusters considered. Based on the NBO analyses, the magnetic moments including the total (TMMs) and local (LMMs) values are defined as the difference between the numbers of spin-up and spin-down electrons occupying the molecular/atomic orbitals of the cluster/atom.

## 3. Results and discussion

### 3.1. Geometrical structures

**Fig. 1:**
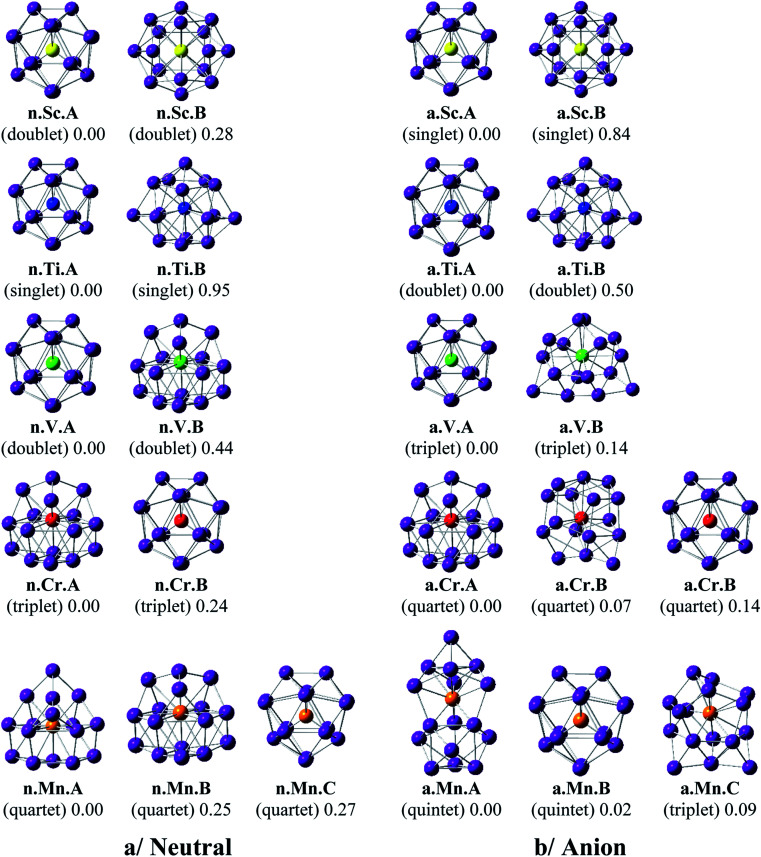
Geometry, relative energy, and spin state (in the bracket) of the most stable isomers Ge_16_M^0/−^, with M = Sc, Ti, V, Cr, and Mn using (U)B3PW91/6-311+G(d) optimizations.

**Fig. 2:**
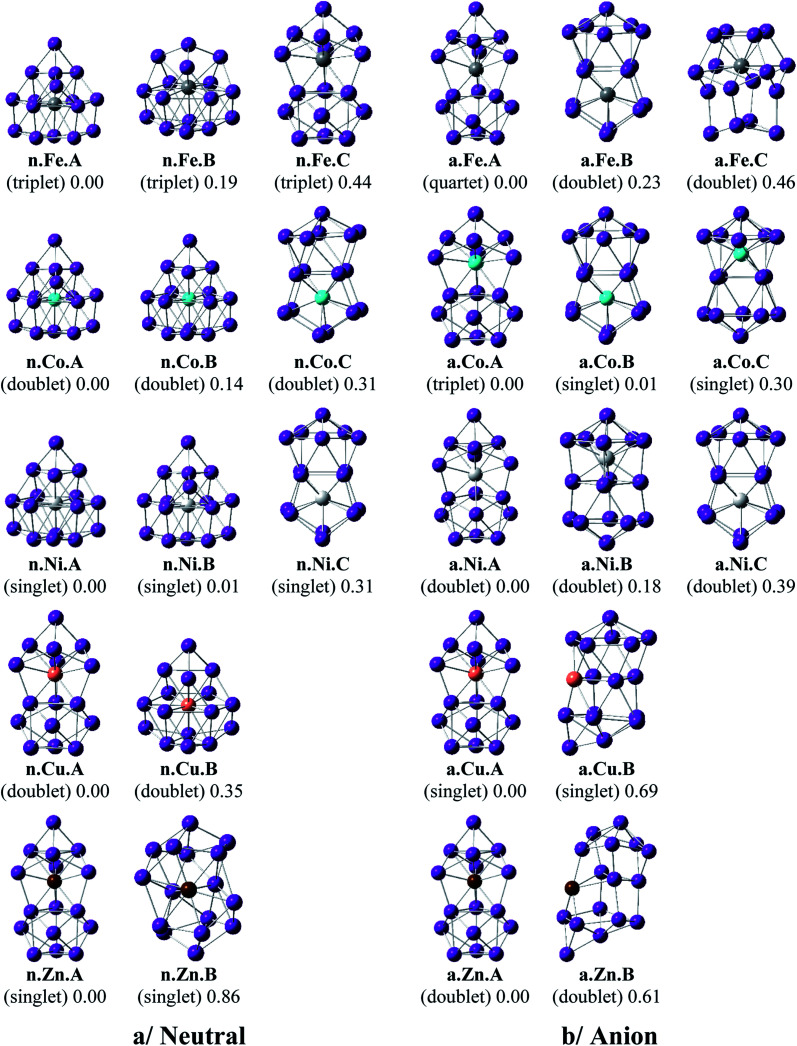
Geometry, relative energy, and spin state (in the bracket) of the most stable isomers Ge_16_M^0/−^, with M = Fe, Co, Ni, Cu, and Zn using (U)B3PW91/6-311+G(d) optimizations.

Shapes of the optimized equilibrium structures of both neutral and anionic series of Ge_16_M clusters, their spin states, and DFT relative energies are shown in Fig. 1 and 2. Because of the large number of isomers located on the potential energy surface of each cluster, only some low lying isomers whose relative energies are close to the corresponding ground state structure are presented for each dopant M.

As for a convention, a **X.M.Y** label is used to denote the isomers considered, in which **X** = **n** and **a** stand for a neutral and anionic state, respectively, **M** = Sc, Ti, V, Cr, Mn, Fe, Co, Ni, Cu, and Zn, and **Y** = **A**, **B**, **C**… refers to the different isomers with increasing relative energy. Thus, **X.M.A** invariably refers to the lowest-energy isomer of the **X.M** series.

The main characteristics of the geometrical features can briefly be summarized as follows:

For M = Sc, Ti and V, our calculated results are in good agreement with the previous studies.^36,41^ The most stable Ge_16_M in both neutral and anionic states consistently prefer a FK structure in which the dopant atom is endohedrally located at the central position of the FK Ge_16_ cage. Remarkably, the lowest-lying isomers of both isoelectronic Ge_16_Sc^−^ anion and Ge_16_Ti neutral are much more stable than the next isomers with large relative energy gaps of 0.84 and 0.95 eV, respectively. The most stable Ge_16_V in both neutral and anionic states still retain the FK form, but their relative energy gaps decrease to <0.5 eV. Besides, the low-lying isomers of Ge_16_V^−^ anion are found to exist in the triplet state.

For Ge_16_Cr, the FK is no longer the most stable form, as they are 0.24 and 0.14 eV higher in energy than the neutral and anion, respectively, of another endohedral structure in which the Cr atom is encapsulated in a *C*_3v_ cage. Ge_16_Cr also favors high spin multiplicity, corresponding to the triplet and quartet states for the neutral and anion, respectively.

The three lowest lying isomers of the neutral Ge_16_Mn prefer endohedrally doped structures and are stable in a quartet state. n.Mn.A becomes 0.25 eV more stable than the other cage n.Mn.B. In the anionic state, however, a competition in energy among three most stable isomers emerges with relative energy gaps of <0.1 eV. Particularly, the a.Mn.A, constructed from fusion of two Ge_10_ in which the endohedral Ge atom, or the Ge atom at the vertex of the lower Ge_10_ block, is substituted by the Mn atom,^48^ and the FK a.Mn.B, are energetically degenerate within a small energy difference of only 0.02 eV, and both of them have a magnetic moment of 4 *μ*_B_, arising from a quintet spin state. Remarkably, the geometry of a.Mn.A is also retained as the most stable one for all remaining Ge_16_M^−^ anions, with M being Fe, Co, Ni, Cu and Zn, despite a competition in energy in Ge_16_Co^−^ where the triplet state a.Co.A is only 0.01 eV lower in energy than the singlet a.Co.B. Accordingly, both Co derivatives a.Co.A and a.Co.B are energetically degenerate.

The shapes of the low lying isomers of the neutral Ge_16_Fe, Ge_16_Co, and Ge_16_Ni are similar to that of the Ge_16_Mn. However, optimization calculations indicate that the two endohedral isomers n.Ni.A and n.Ni.B of Ge_16_Ni are again practically degenerate with a negligible energy gap of 0.01 eV. Finally, the lowest-lying isomers of Ge_16_Cu and Ge_16_Zn exhibit the same shape in both neutral and anionic states.

### 3.2. Stabilities

In order to probe the inherent thermodynamic stability of the Ge_16_M clusters considered, their average binding energies (*E*_b_) are examined and compared to those of the relevant pure germanium clusters Ge_17_ in both neutral and anionic states. The *E*_b_ values of the Ge_16_M clusters can conventionally be defined in eqn (1) and (2):

1 *E*_b_(Ge_16_M) = [16*E*(Ge) + *E*(M) − *E*(Ge_16_M)]/17

2 *E*_b_(Ge_16_M^−^) = [15*E*(Ge) + *E*(Ge^−^) + *E*(M) − *E*(Ge_16_M^−^)]/17

where *E*(Ge), *E*(Ge^−^), and *E*(M), are the total energies of the Ge-atom, the anion Ge^−^, and the M-atom, respectively. *E*(Ge_16_M) and *E*(Ge_16_M^−^) are the total energies of the neutral and anionic of Ge_16_M, respectively.

Similarly, for the neutral Ge_17_ and anionic Ge_17_^−^, the *E*_b_ can be defined by eqn (3) and (4), respectively, as follows:

3 *E*_b_(Ge_17_) = [17*E*(Ge) − *E*(Ge_17_)]/17

**Fig. 3:**
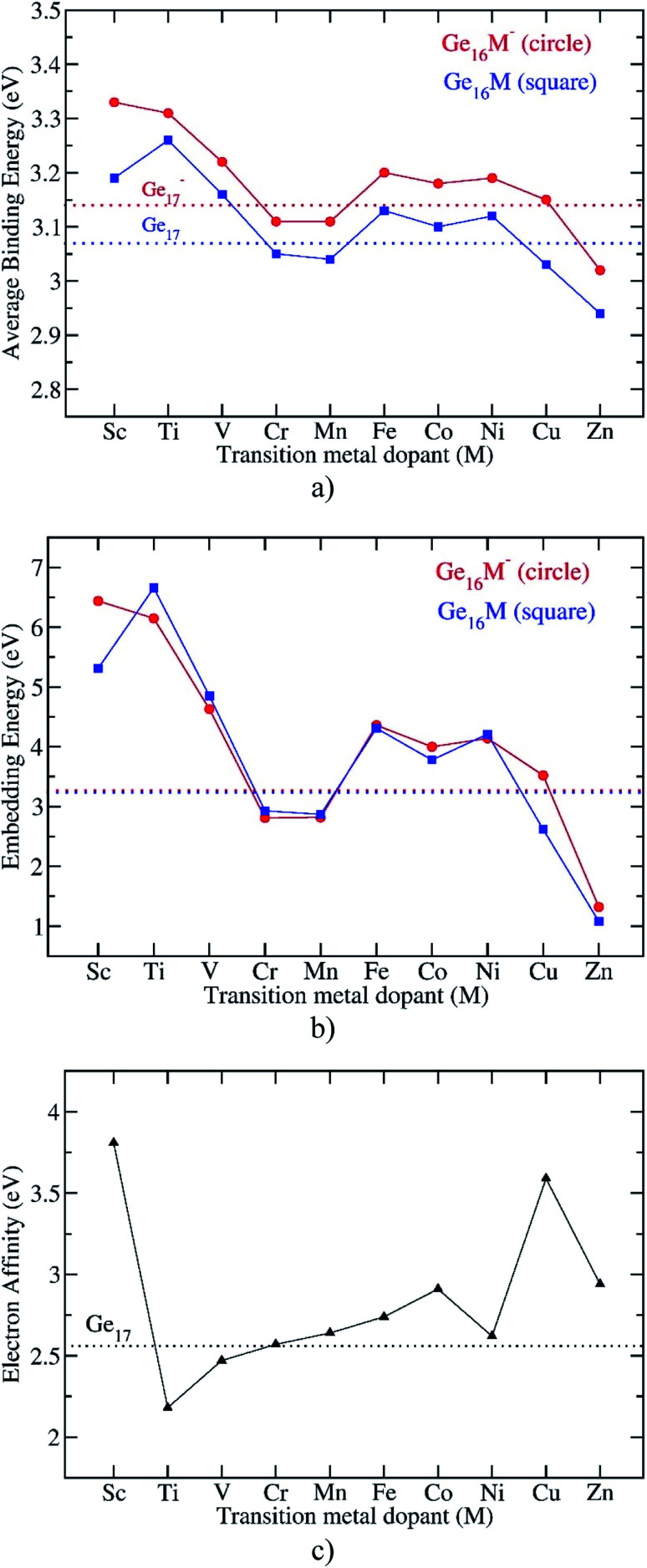
Evolution of the average binding energy, embedding energy, and electron affinity of the Ge_16_M clusters considered. Values are obtained from (U)B3PW91/6-311+G(d) + ZPE computations.

4 *E*_b_(Ge_17_^−^) = [16*E*(Ge) + *E*(Ge^−^) − *E*(Ge_17_^−^)]/17

where *E*(Ge_17_) and where *E*(Ge_17_^−^) are the total energies of the pure neutral and anionic Ge_17_, respectively, that were reported in the previous studies.^10,11^ All these energy values are obtained from B3PW91/6-311+G(d) + ZPE calculations and the plots of *E*_b_ depicted in Fig. 3a illustrate their evolution. The trends of *E*_b_ values in both neutral and anionic Ge_16_M are quite similar to each other. In comparison to the *E*_b_ value of Ge_17_, the *E*_b_ values of Ge_16_M are higher when the M dopant goes from Sc to V, then decrease to lower values with M being Cr and Mn. As M goes from Fe to Ni, the *E*_b_ values of Ge_16_M^0/−^ return to be higher than that of Ge_17_^0/−^. For M = Cu, the *E*_b_ value of the neutral Ge_16_Cu is approximately equal to that of Ge_17_ whereas that of the anionic Ge_16_Cu^−^ becomes lower than the corresponding value of Ge_17_^−^. Finally, Ge_16_Zn takes the lowest *E*_b_ values in both neutral and anionic states. These calculated results prove that a doping of the first-row transition metal M, except for Cr, Mn, Cu and Zn, into Ge_16_ enhances the cluster stability as compared to the pure germanium clusters Ge_17_ in both neutral and anionic states. Remarkably, the neutral Ge_16_Ti and anionic Ge_16_Sc^−^, which possesses each 68 valence electrons, reveal the highest *E*_b_ values as compared to the remaining Ge_16_M counterparts.

To reinforce the above findings, we further examine the embedding energy (EE) of the clusters considered. Embedding energy is defined as the energy gained in incorporating a M-dopant into the Ge_16_ hosts and defined by eqn (5):

5 EE(Ge_16_M^0/−^) = *E*(Ge_16_^0/−^) + *E*(M) − *E*(Ge_16_M^0/−^)

where *E*(Ge_16_^0/−^) are the total DFT energies of the neutral and anionic Ge_16_ clusters, respectively. These total energies are calculated for the ground states of the pure clusters Ge_16_^0/−^ which were previously reported.^10,11^Fig. 3b indicates that both Ge_16_Ti and Ge_16_Sc^−^ are really characterized by the highest EE values for the neutral Ge_16_M and anionic Ge_16_M^−^ clusters, respectively. These predictions are in good agreement with the *E*_b_ values mentioned above, and it can thus be concluded from these observations that an enhanced thermodynamic stability is established for both isoeletronic Ge_16_Ti and Ge_16_Sc^−^ species.

The *E*_b_ values of all anionic Ge_16_M^−^ and pure Ge_17_^−^ clusters are obviously higher than those of the neutral counterparts, as shown in Fig. 3a. An examination of the computed adiabatic electron affinities (EA) of neutral Ge_16_M, in comparison to that of Ge_17_, can thus give us a better insight into this feature. As shown in Fig. 3c, except for Ti and V, the first-row transition metal doped germanium clusters Ge_16_M have the larger EA values than that of Ge_17_. When the M-dopant varies from Sc to Zn, the EA of Ge_16_M takes the largest value of 3.8 eV at Ge_16_Sc, then decrease sharply and reaches the lowest value of 2.2 eV at the next member Ge_16_Ti. Then, the EA value gradually increases as M-dopant goes from Ti to Co, then slightly decreases at Ge_16_Ni before strongly increases at the coinage metal Ge_16_Cu and finally decreases again at Ge_16_Zn.

The large EA of Ge_16_Sc can be interpreted in the same way as that applied to the neutral Al_13_, which is well-known for its very large electron affinity exceeding that of halogen atoms and has thus been named as a superhalogen.^50^ Similarly, the neutral Ge_16_Sc, as formed from the detachment of one electron from the closed-shell structure of the anion Ge_16_Sc^−^ possessing an enhanced thermochemical stability, also has a very large electron affinity. As stated above, calculations reveal that the EA of Ge_16_Sc amounts to 3.8 eV, which is even larger than that of 3.6 eV of Al_13_.^51^ In contrast to Ge_16_Sc, the following neutral member Ge_16_Ti, which is stabilized by a closed shell filled by 68 valence electrons in a singlet state, exhibits the smallest EA value due to the low stability of the corresponding anion. In this context, Ge_16_Sc can be considered as a superhalogen.

The enhanced stability of both isoelectronic Ge_16_Ti and Ge_16_Sc^−^ FK structures can be rationalized by examining their MO pictures under the viewpoint of the electronic shells in a Jellium model (JM),^52^ which is successfully applied to clarify the stability of various structural motifs of atomic clusters in previous studies, particularly those based on Group IVa atoms.^48,53,54^ According to this simple model, the valence electrons are freely movable in a simple mean-field potential constructed by the nuclei of atoms; the valence electrons fill the orbitals following the pattern of atomic orbitals (AO) as [1S^2^ 1P^6^ 1D^10^ 2S^2^ 1F^14^ 2P^6^ 1G^18^ 2D^10^…] corresponding to the numbers of electrons of 2, 8, 18, 20, 34, 40, 58 and 68, *etc.*, that emerge as the magic numbers consistent with a complete filling of the successive electronic shells. As a consequence, a cluster that possesses a valence electron number belonging to this magic number series is able to attain an enhanced thermodynamic stability.

**Fig. 4:**
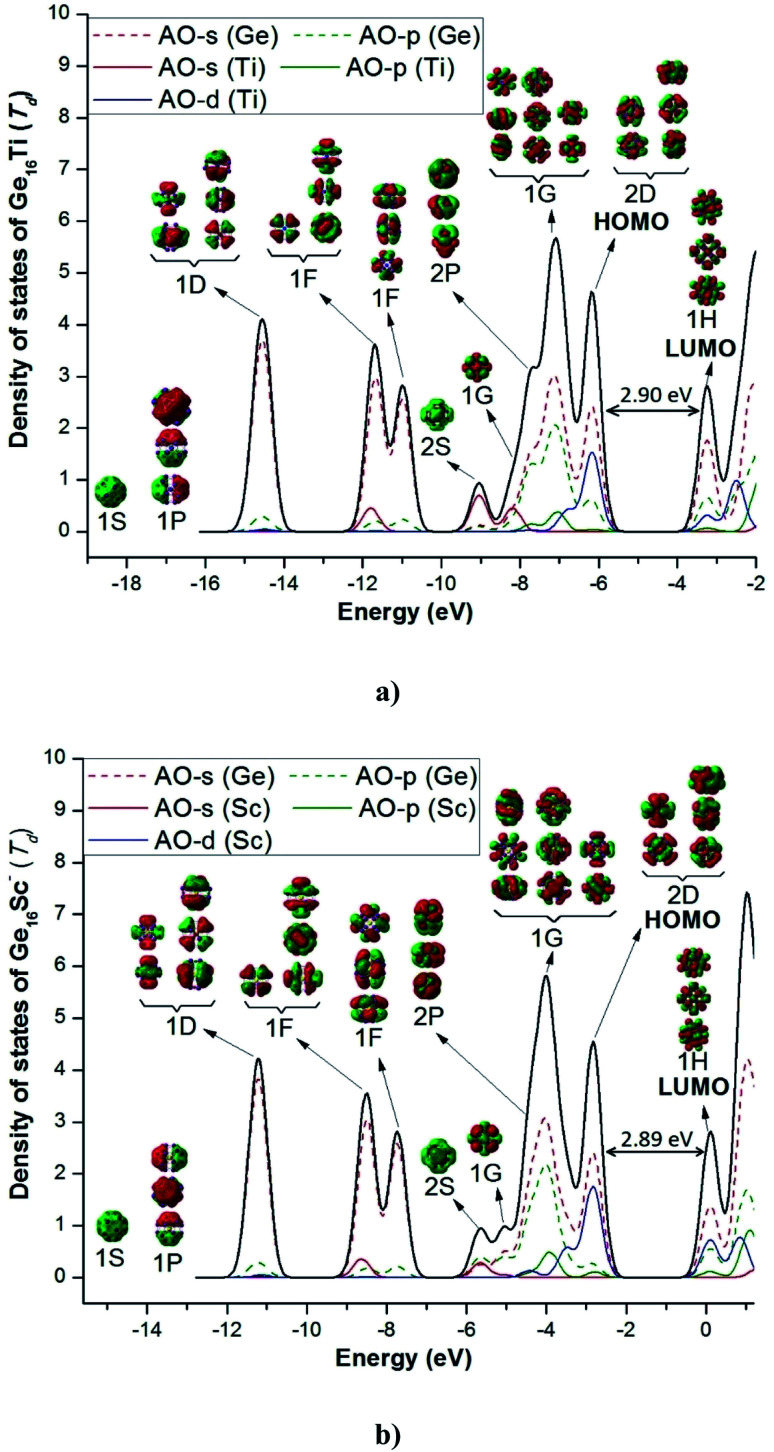
Total (DOS) and partial (pDOS) densities of state of (a) Ge_16_Ti and (b) Ge_16_Sc^−^. Shapes of orbitals of clusters are obtained from B3PW91/6-311+G(d) calculations.

Both Ge_16_Ti and Ge_16_Sc^−^ FK's are characterized by a closed electronic shell configuration with the magic number of 68 valence electrons. The shapes of the relevant MOs and their energy levels, as illustrated in Fig. 4, reveal the great similarity between their densities of states (DOS) and thus prove that they have similar electronic structure as well as the thermochemical stability. The 68 valence electrons of each cluster are distributed in the following orbital configuration:[(1A_1_)^2^ (1T_2_)^6^ (1E)^4^ (2T_2_)^6^ (2A_1_)^2^ (3T_2_)^6^ (1T_1_)^6^ (3A_1_)^2^ (4A_1_)^2^ (4T_2_)^6^ (2T_1_)^6^ (5T_2_)^6^ (2E)^4^ (3E)^4^ (6T_2_)^6^].

This corresponds to a sequence of electronic shell model as:[1S^2^ 1P^6^ 1D^10^ 1F^14^ 2S^2^ 1G^2^ 2P^6^ 1G^16^ 2D^10^]

For both clusters, the lowest-lying MOs include an s-type valence orbital of the 1S and three p-type orbitals of the 1P subshells. The MOs of both 1D and 1F subshells are mainly composed of s-AO of Ge and the remaining AOs of both germanium and the transition metal dopant with much smaller contributions. For the neutral Ge_16_Ti, the MO of 2S subshell are principally formed by the s-AO(Ti), whereas the MO of 2S of anion Ge_16_Sc^−^ are constructed by interaction between s-AO(Sc) and p-AOs(Ge). The MOs of 2P and 1G subshells are constructed by a combination of both s- and p-AOs of Ge-atoms. Finally, the 2D subshell is composed of both s- and p-AO of Ge and d-AO of the metal impurity. In general, the electronic configuration of the both neutral Ge_16_Ti and anion Ge_16_Sc^−^ basically satisfies the electronic shell model of [1S^2^ 1P^6^ 1D^10^ 1F^14^ 2S^2^ 1G^2^ 2P^6^ 1G^16^ 2D^10^] and makes them the enhanced stability species with a magic number of 68 valence electrons.

**Fig. 5:**
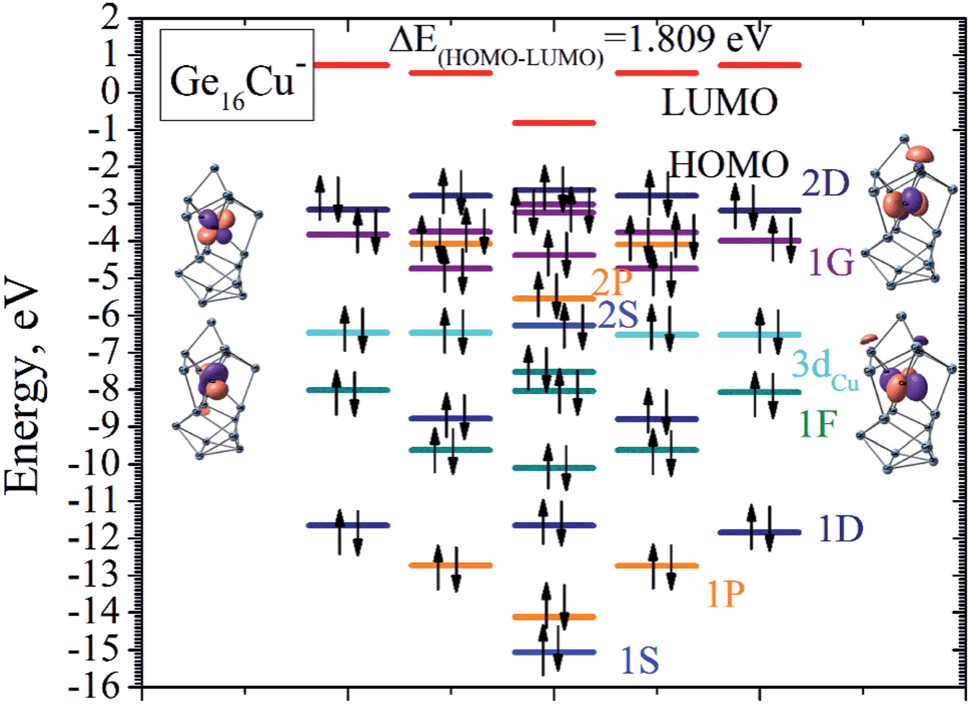
A MO energy diagram of the Ge_16_Cu^−^ anionic cluster and the four 3d orbitals of the Cu dopant atom in the insert. Black arrow represents electron. Bright blue lines represent the 1S and 2S shell orbitals; orange for the 1P and 2P shell orbitals; dark blue for the 1D and 2D shell orbitals; green for the 1F shell orbitals; violet for the 1G shell orbitals; cyan lines for the 3d orbital of Cu and red lines represent HOMO of the cluster.

Remarkably, in addition to the finding of Ge_16_Sc superhalogen, calculations reveal that the ground state of the anion Ge_16_Cu^−^ also has a closed electronic shell and high stability as compared to its neutral form, and being much more stable than the second isomer with a large relative energy gap of 0.69 eV. This is caused by the fact that the neutral Ge_16_Cu has a large electron affinity of 3.6 eV, again approximate to those of the chlorine atom and Al_13_ mentioned above. Accordingly, Ge_16_Cu can also be considered as a superhalogen. The large EA of Ge_16_Cu can be interpreted based on MO approaches. Of the Ge_16_M clusters in both neutral and anionic forms, the electronic structure of the anion Ge_16_Cu^−^ can be considered as a closed-shell by 68 electrons in the pool of valence electrons of the whole cluster such as in the case of Ge_16_Sc^−^ and Ge_16_Ti even though it possesses a non-spherical like geometry. This could be rationalized by considering the following fact. Each Ge delocalizes four electrons whereas the Cu dopant delocalizes three of its eleven 4s^1^3d^10^ valence electrons including one 4s and two 3d into the pool, and while the added electrons are also delocalized in the shell of the entire cluster, the eight remaining 3d electrons are localized in four 3d orbitals of the central Cu. The reason for such a behaviour of the Cu atom in the Ge_16_Cu^−^ anion is that it is located at the trigonal-prismatic hole formed by six nearest Ge atoms. Following the ligand field effects induced by the trigonal-prismatic coordination within a *C*_3v_ point group of the whole Ge_16_Cu^−^ cluster, the five degenerate 3d AOs of the Cu atoms split into three groups of 
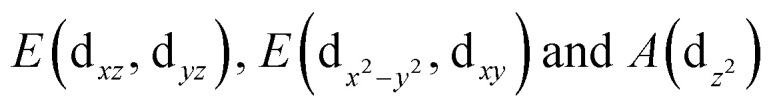
 irreducible representations. While the two former groups have lower energies, the latter possessing higher energy matches the energies of the valence AOs of the Ge neighboring atoms, and thereby combine well with them to form shell MOs of the resulting cluster. Based on shapes and relative energies of the MOs, we can now assign the electronic energy levels of the anion Ge_16_Cu^−^ as [1S^2^ 1P^6^ 1D^6^ 1F^6^ 1D^4^ 1F^8^ 3d_Cu_^8^ 2S^2^ 2P^2^ 1G^6^ 2P^4^ 1G^10^ 2D^4^ 1G^2^ 2D^6^]. The images of the 3d_Cu_ orbitals are represented in the insert of Fig. 5.

### 3.3. Electron shell of 68 valence electrons and Jahn–Teller distortion

Let us now describe in some detail the electronic configurations of the neutral Ge_16_Ti as well as the anionic Ge_16_Sc^−^ that possess FK shape in *T*_d_ symmetry. Each Ge atom in Ge_16_Ti contributes four valence electrons, whereas the Sc and Ti atom contribute three and four, respectively, to its cluster shell. The number of valence electrons contributed by the constitution atoms to the cluster shell amounts to 68 that occupy thus 34 shell MOs. As described in Section 3.2 above, the electronic configuration of this cluster can be written as [1S^2^ 1P^6^ 1D^10^ 2S^2^ 1F^14^ 2P^6^ 1G^18^ 2D^10^] in which the HOMO is the 2D shell and the LUMO is the 1H. In a *T*_d_ point group, the D shell orbitals of either Ge_16_Ti or Ge_16_Sc^−^ split into 2-fold and 3-fold degenerate orbitals, corresponding to an E irreducible representation and a T irreducible representation, respectively, or it could be written as D = E + T. The F shell orbital of both Ge_16_Ti and Ge_16_Sc^−^ split to 1-fold and 3-fold and 3-fold degenerate orbitals, namely F = T + T + A. In the same vein, the G shell orbital splits to 1-fold and 3-fold, 3-fold and 2-fold degeneracy orbitals, namely G = A + 2T + E. Therefore, the electron shell configuration of each cluster could be written as follows: [1S^2^ 1P^6^ 1D_2-folds_^4^ 1D_3-folds_^6^ 2S^2^ 1F_3-folds_^6^ 1F_3-folds_^6^ 1F_1-fold_^2^ 2P^6^ 1G_1-fold_^2^ 1G_3-folds_^6^ 1G_3-folds_^6^ 1G_2-folds_^4^ 2D^10^].

**Fig. 6:**
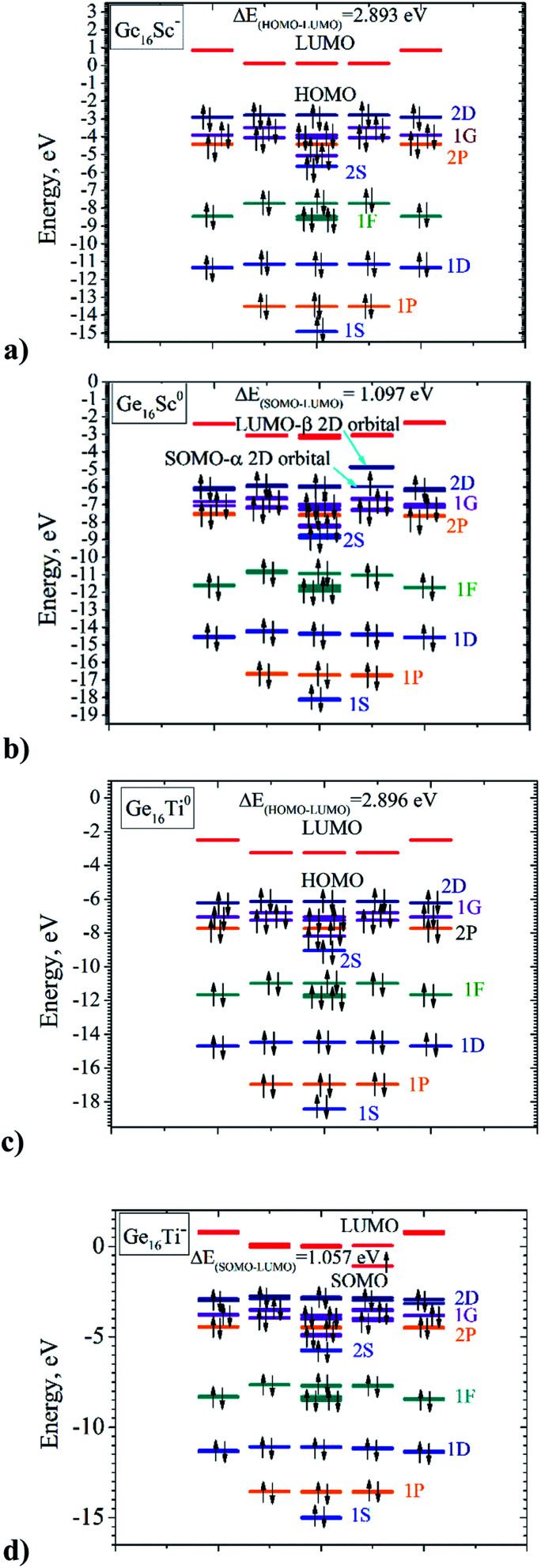
MO diagrams of (a) Ge_16_Sc^−^ anion; (b) Ge_16_Sc^0^ neutral; (c) Ge_16_Ti^0^ neutral; and (d) Ge_16_Ti^−^ anion. Black arrow represents electron. Bright blue lines represent the 1S and 2S shell orbitals; orange for the 1P and 2P shell orbitals; dark blue for the 1D and 2D shell orbitals; green for the 1F shell orbitals; violet for the 1G shell orbitals, and red lines represent for HOMO of the clusters.

A closer look at the shapes of shell MOs shows that the ordering of some shell MOs alters, and the shell electron configuration of the cluster becomes: [1S^2^ 1P^6^ 1D_2-folds_^4^ 1D_3-folds_^6^ 1F_1-fold_^2^ 1F_3-folds_^6^ 1F_3-folds_^6^ 2S^2^ 1G_1-fold_^2^ 2P^6^ 1G_3-folds_^6^ 1G_3-folds_^6^ 1G_2-folds_^4^ 2D_2-folds_^4^ 2D_3-fold_^6^] as this can be seen in Fig. 6a and c.

Nevertheless, geometry optimizations reveal that only FK structures with a closed shell filled by 68 valence electrons, including neutral Ge_16_Ti and anionic Ge_16_Sc^−^ clusters, possess a *T*_d_ high symmetry whereas the remaining FK structures, which enclose more or less than 68 valence electrons, exist at lower symmetry. Such a geometrical distortion can be understood by the Jahn–Teller effect. Let us first examine the open-shell neutral Ge_16_Sc having 67 valence electrons due to the fact that the HOMO of the anion Ge_16_Sc^−^ is a 2D shell orbital; the latter has the value of azimuthal quantum number *l* equal to 2 and a 5-fold degeneracy. In the *T* point group, the 2D shell orbitals are reduced to the E + T irreducible representations in which the T orbitals, or the 2D_three-fold_ levels are higher energetically. As an electron is removed from such a HOMO, namely the 2D_three-fold_ of the anionic Ge_16_Sc^−^, to form the neutral Ge_16_Sc, the corresponding SOMO is triply degenerate and the *T*_d_ structure of the Ge_16_Sc neutral is subjected to a distortion to a *C*_3v_ group, accompanying a splitting of the T MOs, or the 2D_three-fold_ one, into E + A_2_ orbitals, and the A_2_ orbital is now singly occupied (*cf.*Fig. 6b).

In the case of 69 electrons of the Ge_16_Ti^−^ anion, it is worth to note that the LUMO of the Ge_16_Ti neutral cluster corresponds to a 1H shell orbital which has the value of azimuthal quantum number *l* of 5 and a 11-fold degeneracy. In the *T* point group, the 1H shell is reduced into E + 3T irreducible representations. In going from the neutral Ge_16_Ti to the anionic Ge_16_Ti^−^, the incoming electron fills in one of the degenerate LUMO of T representation, which thus causes a distortion of the *T*_d_ structure of the Ge_16_Ti neutral, again to a *C*_3V_ structure of the resulting Ge_16_Ti^−^ anion, accompanying with a splitting of the T MOs into E + A_2_ orbitals, of which the A_2_ orbital accommodates the unpaired electron (*cf.*Fig. 6d).

It is also worth mentioning that the vertical electron detachment energy between the neutral Ge_16_Sc and its anion at the geometrical structure of the anion which has 68 electrons, amounts to 3.95 eV, whereas the adiabatic detachment energy is 3.81 eV. Thus, the energy gain of the Ge_16_Sc neutral upon distortion is 0.14 eV. Similarly, the energy gain of Ge_16_Ti^−^ due to a distortion from *T*_d_ to *C*_3v_ symmetry is calculated at a value of 0.23 eV. Such a small but significant amount of energy originates in an intrinsic instability of the T^1^ or the 2D_three-fold_^1^ electron configuration of the HOMOs of Ge_16_Sc, as well as the T^1^ or 1H_three-fold_^1^ configuration of the LUMOs of Ge_16_Ti^−^, all in *T*_d_ symmetry. The latter are therefore distorted to *C*_3v_ point group yielding such an energy gain of the system.

The SOMO–LUMO gap of 1.1 eV in the 67 electrons neutral Ge_16_Sc is very close to the SOMO–LUMO gap in the 69 electrons of anionic Ge_16_Ti^−^. This reflects the fact that both systems undergo a comparable symmetry reduction when going from the Ge_16_Sc^−^ to Ge_16_Sc as well as from Ge_16_Ti to Ge_16_Ti^−^, as this is illustrated in Fig. 6b and d. In summary, two forces are combined in the formation of the *C*_3v_ structures of 67-electron Ge_16_Sc neutral and 69 electron Ge_16_Ti^−^ anion: the major intrinsic stability of the 68 electron Ge_16_Sc^−^ and Ge_16_Ti counterparts which favor a *T*_d_ symmetry, and the reorganization energy gained during the symmetry lowering from *T*_d_ to *C*_3v_ point group. This also makes the average binding energy of Ge_16_Sc^−^ much higher than that of its neutral, while the average binding energy of Ge_16_Ti^−^ is only slightly higher.

### 3.4. Spin magnetic moments

**Fig. 7:**
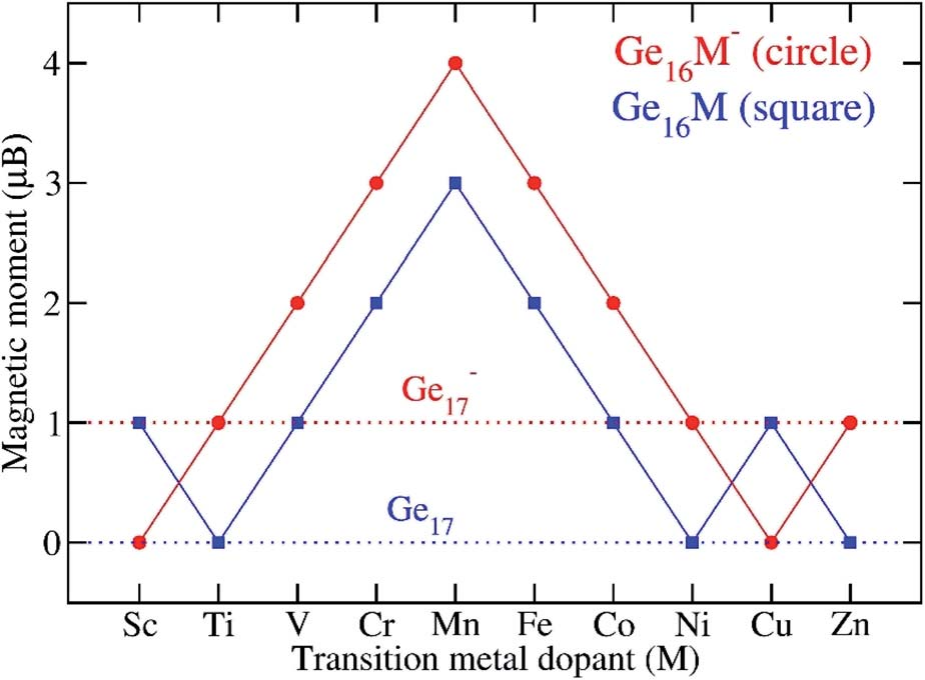
Total spin magnetic moment (*μ*_B_) of Ge_16_M in comparison with Ge_17_ at both neutral and anionic states.

It is typical that when a metallic cluster is doped by a transition metal atom, the outer-most orbitals of the impurity including d and s shell can combine with the valence orbitals of the host to form shell orbitals of the resulting doped clusters. For the investigated Ge_16_M, as each Ge atom delocalizes its 4 valence electrons, the corresponding Ge_16_M^−^ anion can approach 68 electrons if the metal dopant could delocalize its 3 valence electrons. The remaining valence electrons of the metal will combine to build up its magnetic moment. Fig. 7 shows the change in the total spin magnetic moment (TMM) value of Ge_16_M in both neutral and anionic states as M goes from Sc to Zn. The perception of how the total and local spin magnetic moments of the clusters arise can be confirmed in considering the calculated total and local spin magnetic moments on each constituent atoms of the clusters that are listed in Table 1. The total spin magnetic moment of the Ge_16_M^−^ anion increases steadily from 1 *μ*_B_ for the Ti dopant to 4 *μ*_B_ for the Mn dopant, then decreases to 1 *μ*_B_ for the Ni dopant. For the Ge_16_M^−^ anions, their magnetic moments are mostly held on the transition metal atoms, being 0.4, 1.5, 2.9, 3.1, 2.2, and 1.4 for the Ti, V, Cr, Mn, Fe, and Co dopants, respectively. Exceptions include the Ge_16_Ni^−^ and Ge_16_Zn^−^ in which the total magnetic moments are delocalized all over the entire skeleton.

Total spin magnetic moment (TMM, *μ*_B_) of Ge_16_M and local spin magnetic moment (LMM, *μ*_B_) of each atom at (a) neutral and (b) anionic states. Atom number and corresponding Cartesian coordinates of each atom in Ge_16_M are given in the Table S1 of the ESI

**Table:** 

(a)							
Atom	Ge_16_Sc	Ge_16_V	Ge_16_Cr	Ge_16_Mn	Ge_16_Fe	Ge_16_Co^−^	Ge_16_Cu
Ge (1)	0.0	0.1	−0.3	0.0	0.1	0.0	0.0
Ge (2)	0.0	0.0	0.0	0.0	0.0	0.0	0.0
Ge (3)	0.1	0.0	0.0	0.0	0.0	0.0	0.1
Ge (4)	0.1	−0.1	0.0	0.0	0.0	0.0	0.1
Ge (5)	0.1	0.0	−0.1	0.0	0.1	0.0	0.0
Ge (6)	0.0	0.0	0.0	0.0	0.0	0.0	0.0
Ge (7)	0.0	0.0	0.0	0.0	0.0	0.0	0.1
Ge (8)	0.0	0.0	0.0	0.0	0.0	0.0	0.1
Ge (9)	0.0	0.0	0.0	0.0	0.0	0.0	0.0
Ge (10)	0.3	0.1	0.0	0.0	0.0	0.0	0.0
Ge (11)	0.0	−0.1	−0.1	0.0	0.1	0.0	0.0
Ge (12)	0.0	0.0	−0.1	0.1	0.1	0.0	0.4
Ge (13)	0.1	0.1	0.0	0.1	0.1	0.1	0.1
Ge (14)	0.0	0.0	0.0	0.0	0.0	0.0	0.1
Ge (15)	0.0	0.0	0.0	0.1	−0.1	0.0	0.0
Ge (16)	0.1	−0.1	0.0	0.0	−0.1	0.0	0.0
M (17)	0.2	1.0	2.6	2.7	1.7	0.9	0.0
TMM	1.0	1.0	2.0	3.0	2.0	1.0	1.0

**Table:** 

(b)									
Atom	Ge_16_Ti^−^	Ge_16_V^−^	Ge_16_Cr^−^	Ge_16_Mn^−^	Ge_16_Fe^−^	Ge_16_Co^−^	Ge_16_Ni^−^	Ge_16_Zn^−^	Ge_17_^−^
Ge (1)	0.1	−0.1	0.0	0.1	0.1	0.1	0.0	0.0	0.0
Ge (2)	0.0	0.0	0.1	0.0	0.1	0.0	0.0	0.1	0.1
Ge (3)	0.0	0.1	0.1	0.0	0.0	0.0	0.2	0.1	0.0
Ge (4)	0.0	0.0	0.1	0.0	0.0	0.0	0.0	0.1	0.0
Ge (5)	0.0	0.0	0.0	0.1	0.1	0.0	0.1	0.0	0.0
Ge (6)	0.0	0.0	0.0	0.1	0.1	0.1	0.1	0.0	0.0
Ge (7)	0.0	0.0	0.0	0.0	0.0	0.0	0.0	0.1	0.0
Ge (8)	0.1	0.1	0.0	0.0	0.0	0.0	0.1	0.1	0.1
Ge (9)	0.1	0.0	0.0	0.0	0.0	0.1	0.1	0.0	0.0
Ge (10)	0.0	0.0	0.0	0.1	0.1	0.1	0.0	0.0	0.1
Ge (11)	0.0	0.1	0.0	0.1	0.0	0.0	0.0	0.1	0.1
Ge (12)	0.1	0.0	0.0	0.2	0.2	0.2	0.0	0.1	0.1
Ge (13)	0.1	0.1	−0.1	0.0	0.0	0.0	0.1	0.1	0.0
Ge (14)	0.0	0.1	−0.1	0.0	0.0	0.0	0.1	0.1	0.5
Ge (15)	0.1	0.0	−0.1	0.1	0.1	0.0	0.0	0.1	0.0
Ge (16)	0.0	0.1	0.1	0.1	0.0	0.0	0.0	0.0	0.0
M (17)	0.4	1.5	2.9	3.1	2.2	1.4	0.2	0.0	0.0
TMM	1.0	2.0	3.0	4.0	3.0	2.0	1.0	1.0	1.0

**Fig. 8:**
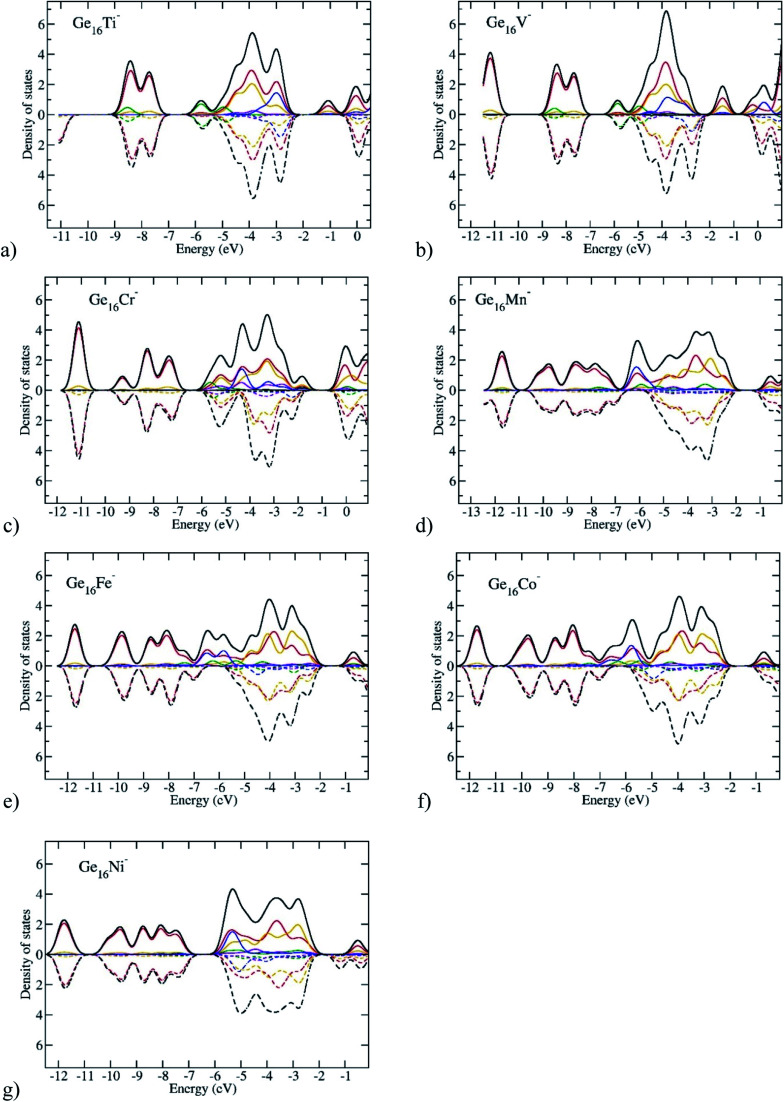
Plots of density of states of Ge_16_TM anionic clusters. The red lines represent for the s states of Ge, the orange for the p states of Ge, the green for the s state of TM, the violet for the p states of TM, the blue for the d states of TM and the black lines represent for the total density of states. Solid lines represent for alpha spin states, and dashed lines for beta spin states.

In order to gain more insight into the spin magnetic behavior of Ge_16_M clusters, along with Table 1 listing total and local spin magnetic moments, the total density of states (TDOS) and partial density of states (PDOS) of the anionic Ge_16_M^−^ clusters from the Ti to Ni dopants are plotted in Fig. 8. Spin-up and spin-down densities of states are plotted separately on the same graph for each of the clusters. It has been stated that the relative shift between the spin-up and spin-down bands indicates the degree of spin-exchange splitting; the larger the shift of DOS bands, the larger the magnetic moment of the cluster.^55^ As we glance at Fig. 8, we can realize that the shift is large for the Ge_16_M^−^ (M = Cr, Mn, Fe, Co) clusters suggesting that they possess high spin magnetic moments, while it is slight in the cases of Ge_16_Ti^−^ and Ge_16_Ni^−^ clusters.

The spin magnetic moment of the whole cluster is mainly created by the unpaired electrons, and the spin magnetic moments localized on the metal dopant arise from the density of unpaired electron contained in its orbitals or the difference in partial density of α- and β-electrons of the M atom. For the Ti and V clusters (Fig. 8a and b), the α-HOMO states are located at distinctive energy level as compared to the α-, β-inner states, while for the other Ge_16_M, with M being Cr, Mn, Fe, Co, and Ni, the α-HOMO states are situated in almost the same energy region as with the α, and β-inner states. For the DOS of Ge_16_Cr^−^ anion, in the domain of states ranging from −4.3 to −2.0 eV, there is an obvious presence of the density of α-state of d orbitals of Cr (the blue curve) without the β-state counterpart. This information implies that the total magnetic moment of the cluster is mainly dominated by Cr-d states, while Ge-s and Ge-p states make rather a small contribution. A similar argument can be made for the successive clusters. For Ge_16_Mn^−^ in the domain from *ca.* −6.5 to −2.0 eV there are densities of α-states of s- and d-orbitals of Mn (the green and blue curves) without their β-state counterparts; for Ge_16_Fe^−^ in the domain from *ca.* −7.0 to −2.0 eV there are densities of α-states of s- and d-orbitals of Fe (the green and blue curves) without their β-state counterparts; for Ge_16_Co^−^ in the domain from *ca.* −7.0 to −2.0 eV there are densities of α-states of s- and d-orbitals of Co (the green and blue curves) without their β-state counterparts. This observation is in line with the calculated local magnetic moments on the Cr, Mn, Fe, Co atoms which amount to 2.9, 3.1, 2.2 and 1.4 *μ*_B_, respectively. Thus, although the Ge_16_ cage somehow quenches the usually large magnetic moments of free transition metal atoms, the latter property remain substantial in the doped derivatives.

### 3.5. IR spectra

**Fig. 9:**
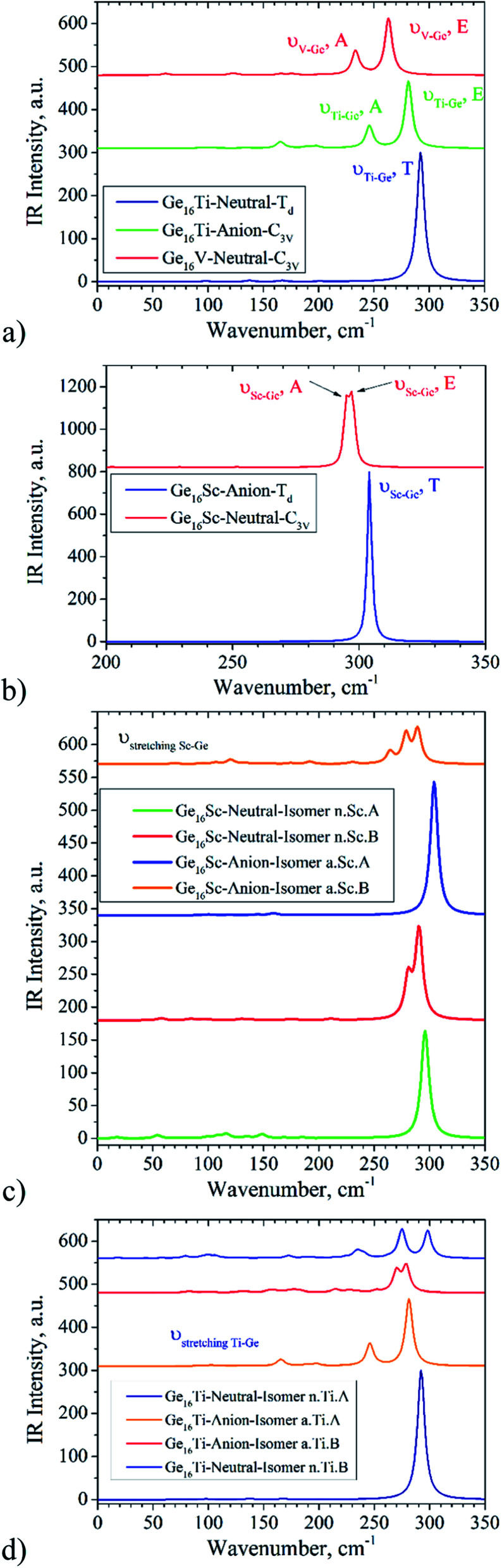
The IR spectra of: (a) Ge_16_Ti^0/−^ and Ge_16_V. The red curve represents for IR spectrum of Ge_16_V neutral, the green curve for Ge_16_Ti^−^ anion, and the dark blue for the Ge_16_Ti neutral; (b) Ge_16_Sc^0/−^ clusters. The red curve represents for the Ge_16_Sc neutral, and the blue for the Ge_16_Sc^−^ anion; (c) the two lowest-energy isomers of the Ge_16_Sc^0/−^; (d) the two lowest-energy isomers of the Ge_16_Ti^0/−^.

As discussed above, several lower-energy structural and spin isomers for each Ge_16_M cluster are considered, and we report herein only the lowest-energy isomers for certain species. In some cases, the energy difference of the most stable isomers is really small that cannot allow us to distinguish the ground state structure. Moreover, no infrared (IR) spectrum of any Ge_*n*_M has been reported both experimentally and theoretically so far. The IR spectra are expected to provide us with a fingerprint for assignment of the cluster in terms of their metallic dopant, especially for the geometrical structure. For the purpose to help for distinguishing low-lying energy isomers, the calculated vibrational spectra of two lowest-lying isomers for some selected Ge_16_M, namely the Ge_16_Sc and Ge_16_Ti in both neutral and anionic forms, are plotted in Fig. 9. The vibrational frequency range goes from 0 to 350 cm^−1^, as no signals are found at higher photon energy. As it could be seen in Fig. 9a and b, the vibrational spectra for the ground state of Ge_16_Sc^−^ anion and Ge_16_Ti neutral, each has 68 valence electrons and is in *T*_d_ symmetry, are relatively simple featuring a highly intense peak, centered at ∼304 and 292 cm^−1^ with *T* degenerate modes, respectively.

Unlike the IR spectra of the ground states, those of the next isomers turn out to be more complicated and characterized by several highly intense peaks in the range of 260 to 300 cm^−1^ (see Fig. 9c and d). The following frequencies can be noted. While for the ground state of Ge_16_Sc neutral, isomer n.Sc.A has *ν*_stretching Sc–Ge_ being of ∼298 cm^−1^, the *ν*_stretching Sc–Ge_ of the next isomer n.Sc.B is of ∼280 and 290 cm^−1^. For the Ge_16_Sc^−^ anion, FK structure a.Sc.A has a *ν*_stretching Sc–Ge_ of ∼304 cm^−1^, while *ν*_stretching Sc–Ge_ of the isomer a.Sc.B is of ∼264, 279 and 289 cm^−1^. Although for Ge_16_Ti, FK structure n.Ti.A only has one peak at 292 cm^−1^, being three-fold degenerate Sc–Ge stretching mode, for the next isomer n.Ti.B, the Sc–Ge stretching modes appear at three frequencies ∼263, 275 and 298 cm^−1^. For the anion Ge_16_Ti^−^, a.Ti.A in distorted FK shape (*C*_3v_) leads its *ν*_stretching Sc–Ge_ being of ∼246 and 281 cm^−1^, which is a two-fold degenerate mode E, while a.Ti.B isomer has a *ν*_stretching Sc–Ge_ being of ∼252, 270 and 279 cm^−1^. The large difference in vibrational spectra for both nearly degenerate isomers and their high intense peaks can be used to assign the ground state structure when they can be generated experimentally and characterized spectroscopically.

By analysis of their IR spectra, a symmetry lowering of the cluster in going down from 68 to 67 as well as going up to 69 valence electrons can be recognized. The IR spectrum of Ge_16_Ti is characterized by a single peak centered at 292 cm^−1^, corresponding to the vibrational modes of Ti atom inside the Ge_16_ cage. Although the cluster has 45 vibrational modes in total, only the vibrational modes of the Ti atom inside the cage are IR active with notably high intensity. Other modes that correspond to deformation of the Ge_16_ cage do not result in significant IR intensity. The stretching modes of the Ti atom inside the Ge_16_ cage belong to the T_2_ irreducible representation. As the cluster receives one electron to form the Ge_16_Ti^−^, the anion is distorted to *C*_3v_ point group and the *ν*(Ti–Ge_16_) T_2_ mode is reduced to the E + A_2_ modes. As this could be seen in Fig. 9, the difference of 22 cm^−1^ between the E and A_2_ modes is relatively significant in view of the low frequency. It is worth noting that for Ge_16_Ti^−^, its A_2_*ν*(Ti–Ge_16_) stretching mode has a lower frequency than the E *ν*(Ti–Ge_16_) stretching. The added electron in the Ge_16_Ti^−^ anion causes the cluster to distort from *T*_d_ to *C*_3v_ point group, giving rise to a lowering of electron density along one of the three *ν*(Ti–Ge_16_) stretching modes.

## 4. Concluding remarks

In the present theoretical study, the geometric and electronic structures, thermodynamic stability, and magnetic properties of the 16-atom germanium clusters doped with the first-row 3d transition metal atoms, Ge_16_M with M = Sc, Ti, V, Cr, Mn, Fe, Co, Ni, Cu, and Zn, in both neutral and anionic states, were investigated using quantum chemical (DFT) methods.

The most stable isomers of Ge_16_M, as M goes from Sc to V, prefer a Frank–Kasper (FK) structure in which the metal dopant is endohedrally encapsulated at the central position of a Ge_16_ FK cage. In particular, both the anionic Ge_16_Sc^−^ and neutral Ge_16_Ti whose electronic shells are filled by a magic number of 68 valence electrons, are characterized by a perfect FK tetrahedral geometry and enjoy an enhanced thermochemical stability with high average binding energies and embedding energies. Their higher stability can be interpreted in terms of the electronic shells of the Jellium model. Analyses of electronic configuration also indicate that the geometric distortions from an FK tetrahedron Ge_16_M having more or less than 68 valence electrons are caused by a Jahn–Teller effect arising from the degenerate frontier orbitals.

Perhaps most interestingly is the result obtained from energy calculations that revealed that both neutrals Ge_16_Sc and Ge_16_Cu emerge as superhalogens, due to a characteristic that each possesses a large electron affinity of 3.8 and 3.6 eV, respectively. These electron affinities exceed the values of halogen atoms and even that of the well-known superhalogen Al_13_ (∼3.6 eV). Moreover, a comprehensive picture of the magnetic behavior is displayed for Ge_16_M clusters at both neutral and anionic states, in which the observed dopant-dependent magnetic moment can be understood by a charge distribution analysis. As M goes from the left to the right side on the first-row transition metal atoms in the Periodic Table, corresponding to Sc to Zn, the total magnetic moment of Ge_16_M first takes a low value at M = Sc and Ti, then increases steadily and reaches the maximum value of 3 *μ*_B_ at M = Mn, before decreasing towards the end of the row due to the fact that these magnetic moments are mostly held on the metal dopants. This result opens up an avenue that a magnetically inert germanium cluster can be induced to a relatively high magnetic moment following doping by a suitable transition metal impurity. Finally, the IR spectra of FK Ge_16_M are simulated as a helpful guide for future experimental assignment of these degenerate ground state clusters.

## Funding information

This work is funded by VinGroup (Vietnam) and supported by VinGroup Innovation Foundation (VinIF) under project code VinIF.2020.DA21.

## Conflicts of interest

There are no conflicts of interest to declare.

## Supplementary Material

RA-012-D1RA08527A-s001
