# Supplementary material for: First-row transition metal doped germanium clusters Ge16M: some remarkable superhalogens
Source: RSC Adv. 2022 May 4;12(21):13487–99. doi: 10.1039/d1ra08527a (PMC9068264; doi:10.1039/d1ra08527a)
Supplement: RA-012-D1RA08527A-s001 [file RA-012-D1RA08527A-s001.pdf]

Electronic Supplementary Material (ESI) for RSC Advances.
This journal is © The Royal Society of Chemistry 2022

# Supplementary Information

# The first-row transition metal doped germanium clusters $Ge_{16}M$: Some remarkable superhalogens

**Huu Tho Nguyen,[a] Ngo Tuan Cuong,[b] Ngo Thi Lan,[c,d] Nguyen Thanh Tung,[c] Minh Tho Nguyen,[e] and Nguyen Minh Tam,[f, g, *]**

[a] *Faculty of Natural Sciences Education, Sai Gon University, 273 An Duong Vuong street, , Ho Chi Minh City, Vietnam*

[b] *Center for Computational Science and Faculty of Chemistry, Hanoi National University of Education, Hanoi, Vietnam*

[c] *Institute of Materials Science and Graduate University of Science and Technology, Vietnam Academy of Science and Technology, 18 Hoang Quoc Viet, Hanoi, Vietnam*

[d] *Institute of Science and Technology, TNU-University of Sciences, Tan Thinh Ward, Thai Nguyen City, Vietnam*

[e] *Institute for Computational Science and Technology (ICST), Quang Trung Software City, Ho Chi Minh City, Vietnam*

[f] *Laboratory of Theoretical and Computational Biophysics, Advanced Institute of Materials Science, Ton Duc Thang University, Ho Chi Minh City, Vietnam. Email: nguyenminhtam@tdtu.edu.vn*

[g] *Faculty of Pharmacy, Ton Duc Thang University, Ho Chi Minh City, Vietnam*

**Table S1.** Cartesian coordinates of the most stable isomers of $Ge_{16}M$ at both neutral and anionic states

| Cluster | Atom number | Element | Cartesian Coordinates |
|---|---|---|---|
| **$Ge_{16}Sc$** neutral | 1 | Ge | 0.00000000 1.53274500 -2.63169500 |
| | 2 | Ge | -1.32739600 -0.76637200 -2.63169500 |
| | 3 | Ge | 0.00000000 -2.68331300 -0.94784200 |
| | 4 | Ge | -1.29485800 -2.28817900 1.59878100 |
| | 5 | Ge | 0.00000000 0.00000000 2.74986000 |
| | 6 | Ge | 1.33419200 2.26547000 1.59878100 |
| | 7 | Ge | 0.00000000 2.94711500 -0.53685100 |
| | 8 | Ge | 1.32739600 -0.76637200 -2.63169500 |
| | 9 | Ge | 2.32381800 1.34165700 -0.94784200 |
| | 10 | Ge | 2.62905100 0.02271000 1.59878100 |
| | 11 | Ge | 1.29485800 -2.28817900 1.59878100 |
| | 12 | Ge | -1.33419200 2.26547000 1.59878100 |
| | 13 | Ge | -2.55227700 -1.47355800 -0.53685100 |
| | 14 | Ge | -2.62905100 0.02271000 1.59878100 |
| | 15 | Ge | -2.32381800 1.34165700 -0.94784200 |
| | 16 | Ge | 2.55227700 -1.47355800 -0.53685100 |
| | 17 | Sc | 0.00000000 0.00000000 0.01008700 |
| **$Ge_{16}Sc^-$** anion | 1 | Ge | 0.90194700 -0.90194700 2.75513800 |
| | 2 | Ge | -0.90194700 0.90194700 2.75513800 |
| | 3 | Ge | -0.90194700 2.75513800 0.90194700 |
| | 4 | Ge | 0.90194700 2.75513800 -0.90194700 |
| | 5 | Ge | -2.75513800 -0.90194700 -0.90194700 |
| | 6 | Ge | -2.75513800 0.90194700 0.90194700 |
| | 7 | Ge | 2.75513800 -0.90194700 0.90194700 |
| | 8 | Ge | 2.75513800 0.90194700 -0.90194700 |
| | 9 | Ge | -0.90194700 -0.90194700 -2.75513800 |
| | 10 | Ge | 0.90194700 0.90194700 -2.75513800 |
| | 11 | Ge | 0.90194700 -2.75513800 0.90194700 |
| | 12 | Ge | -0.90194700 -2.75513800 -0.90194700 |
| | 13 | Ge | -1.63773500 -1.63773500 1.63773500 |
| | 14 | Ge | 1.63773500 1.63773500 1.63773500 |
| | 15 | Ge | 1.63773500 -1.63773500 -1.63773500 |
| | 16 | Ge | -1.63773500 1.63773500 -1.63773500 |
| | 17 | Sc | 0.00000000 0.00000000 0.00000000 |

| | | | |
|---|---|---|---|
| **$Ge_{16}Ti$** neutral | 1 | Ge | 0.89497100 -0.89497100 2.73165100 |
| | 2 | Ge | -0.89497100 0.89497100 2.73165100 |
| | 3 | Ge | -0.89497100 2.73165100 0.89497100 |
| | 4 | Ge | 0.89497100 2.73165100 -0.89497100 |
| | 5 | Ge | -2.73165100 -0.89497100 -0.89497100 |
| | 6 | Ge | -2.73165100 0.89497100 0.89497100 |
| | 7 | Ge | 2.73165100 -0.89497100 0.89497100 |
| | 8 | Ge | 2.73165100 0.89497100 -0.89497100 |
| | 9 | Ge | -0.89497100 -0.89497100 -2.73165100 |
| | 10 | Ge | 0.89497100 0.89497100 -2.73165100 |
| | 11 | Ge | 0.89497100 -2.73165100 0.89497100 |
| | 12 | Ge | -0.89497100 -2.73165100 -0.89497100 |
| | 13 | Ge | -1.58811600 -1.58811600 1.58811600 |
| | 14 | Ge | 1.58811600 1.58811600 1.58811600 |
| | 15 | Ge | 1.58811600 -1.58811600 -1.58811600 |
| | 16 | Ge | -1.58811600 1.58811600 -1.58811600 |
| | 17 | Ti | 0.00000000 0.00000000 0.00000000 |
| **$Ge_{16}Ti^-$** anion | 1 | Ge | -1.28722700 -0.74318100 -2.63887800 |
| | 2 | Ge | 2.26478500 1.30757400 -0.88949500 |
| | 3 | Ge | 1.28722700 -0.74318100 -2.63887800 |
| | 4 | Ge | 0.00000000 1.48636200 -2.63887800 |
| | 5 | Ge | 0.00000000 2.97932700 -0.56219800 |
| | 6 | Ge | -2.26478500 1.30757400 -0.88949500 |
| | 7 | Ge | -1.31072900 2.21986400 1.58439300 |
| | 8 | Ge | -2.57782300 0.02519300 1.58439300 |
| | 9 | Ge | 1.31072900 2.21986400 1.58439300 |
| | 10 | Ge | 0.00000000 0.00000000 2.75227700 |
| | 11 | Ge | 1.26709400 -2.24505700 1.58439300 |
| | 12 | Ge | 2.57782300 0.02519300 1.58439300 |
| | 13 | Ge | -2.58017300 -1.48966400 -0.56219800 |
| | 14 | Ge | 0.00000000 -2.61514800 -0.88949500 |
| | 15 | Ge | -1.26709400 -2.24505700 1.58439300 |
| | 16 | Ge | 2.58017300 -1.48966400 -0.56219800 |
| | 17 | Ti | 0.00000000 0.00000000 0.01902600 |

| | | | |
|---|---|---|---|
| **$Ge_{16}V$** neutral | 1 | Ge | -0.88887700 2.49087000 -1.60784600 |
| | 2 | Ge | 1.63313100 -1.16921000 -2.20621400 |
| | 3 | Ge | 1.74778700 -1.01253900 2.19552000 |
| | 4 | Ge | -1.10058700 2.12283600 1.19630400 |
| | 5 | Ge | 1.45882900 1.54856500 2.09265400 |
| | 6 | Ge | 1.26589200 2.69759400 -0.12855200 |
| | 7 | Ge | 2.79069300 0.31080200 -0.08383500 |
| | 8 | Ge | 1.35121400 1.32139600 -2.30779000 |
| | 9 | Ge | -2.46533800 -1.60641100 -0.61589400 |
| | 10 | Ge | -0.29914600 -2.74572800 -1.39631300 |
| | 11 | Ge | -0.61261700 -2.21176800 1.37363800 |
| | 12 | Ge | -2.55615200 -0.22256300 1.56927000 |
| | 13 | Ge | -0.48068100 0.06952600 3.05412800 |
| | 14 | Ge | -2.75576400 0.96376800 -0.72217200 |
| | 15 | Ge | 1.83354000 -2.35177900 0.07685300 |
| | 16 | Ge | -0.95176400 -0.20857900 -2.48919800 |
| | 17 | V | 0.04151400 0.00448000 -0.00077100 |
| **$Ge_{16}V^-$** anion | 1 | Ge | 0.01092300 -0.05868700 -2.66016100 |
| | 2 | Ge | -0.53036600 -2.55424400 0.98395800 |
| | 3 | Ge | 0.81890400 -2.50568500 -1.54713900 |
| | 4 | Ge | 2.17376400 -1.91656100 0.58153700 |
| | 5 | Ge | 1.13051200 -0.95191000 2.65156800 |
| | 6 | Ge | 2.49001600 0.85222300 0.92098000 |
| | 7 | Ge | 0.58022600 2.86007800 0.46953600 |
| | 8 | Ge | -0.82746400 2.42551100 -1.66145600 |
| | 9 | Ge | 0.29422200 1.55157300 2.59305700 |
| | 10 | Ge | -1.97109400 1.76339600 0.88300300 |
| | 11 | Ge | -2.54058700 0.49609400 -1.62715900 |
| | 12 | Ge | -2.76073600 -0.90864300 0.53953100 |
| | 13 | Ge | 1.74042000 1.90297300 -1.64081500 |
| | 14 | Ge | -1.70895600 -1.98811800 -1.56740700 |
| | 15 | Ge | -1.45617200 -0.42470800 2.62925900 |
| | 16 | Ge | 2.55653000 -0.54418100 -1.58242600 |
| | 17 | V | -0.00019900 0.00123800 0.04749100 |

| | | | |
|---|---|---|---|
| **$Ge_{16}Cr$** neutral | 1 | Ge | -3.18524400 -0.02453000 0.00000000 |
| | 2 | Ge | 1.79598300 -1.54145700 2.69206200 |
| | 3 | Ge | 2.05213300 -1.54128700 0.00000000 |
| | 4 | Ge | -0.21248200 -2.50302900 -1.24999600 |
| | 5 | Ge | -2.18324100 -1.14317000 -1.95206700 |
| | 6 | Ge | -0.24781400 2.33033000 -1.54108400 |
| | 7 | Ge | -0.24781400 2.33033000 1.54108400 |
| | 8 | Ge | 2.03694500 0.79556400 1.34923200 |
| | 9 | Ge | -0.21248200 -2.50302900 1.24999600 |
| | 10 | Ge | -0.23166700 0.16563900 2.79119700 |
| | 11 | Ge | -2.20864100 2.23684100 0.00000000 |
| | 12 | Ge | -2.18324100 -1.14317000 1.95206700 |
| | 13 | Ge | 1.75926500 3.12409500 0.00000000 |
| | 14 | Ge | 1.79598300 -1.54145700 -2.69206200 |
| | 15 | Ge | -0.23166700 0.16563900 -2.79119700 |
| | 16 | Ge | 2.03694500 0.79556400 -1.34923200 |
| | 17 | Cr | -0.44394900 -0.00383300 0.00000000 |
| **$Ge_{16}Cr^-$** anion | 1 | Ge | -2.09091200 -0.12886300 1.55069000 |
| | 2 | Ge | -1.81448000 0.27493100 -3.08512300 |
| | 3 | Ge | -1.77444000 2.55341000 1.79290100 |
| | 4 | Ge | -1.79065600 -2.81382400 1.32716600 |
| | 5 | Ge | -2.10071900 1.40252000 -0.63812800 |
| | 6 | Ge | -2.11213500 -1.25826100 -0.86936200 |
| | 7 | Ge | 0.21048500 -2.76702900 -0.39297900 |
| | 8 | Ge | 0.22924800 2.79384800 0.09113700 |
| | 9 | Ge | 0.19811700 -1.31726900 -2.46772800 |
| | 10 | Ge | 0.23272400 -1.47801800 2.37173200 |
| | 11 | Ge | 0.20991200 1.72183100 -2.20232100 |
| | 12 | Ge | 0.24021600 1.04421400 2.59111000 |
| | 13 | Ge | 2.17038900 -1.89566600 0.86793600 |
| | 14 | Ge | 2.18180000 1.70390500 1.18186600 |
| | 15 | Ge | 2.15404800 0.17397400 -2.09245100 |
| | 16 | Ge | 3.59832900 -0.00911800 -0.02512600 |
| | 17 | Cr | 0.34409800 -0.00077900 -0.00176100 |

| | | | |
|---|---|---|---|
| **$Ge_{16}Mn$** neutral | 1 | Ge | -1.68530427 -1.96119541 1.27433801 |
| | 2 | Ge | -2.21129906 0.45566004 -1.68414802 |
| | 3 | Ge | -1.68530427 -1.96119541 -1.27433801 |
| | 4 | Ge | -2.21129906 0.45566004 1.68414802 |
| | 5 | Ge | -0.44163707 2.49520225 0.00000000 |
| | 6 | Ge | -0.05419419 0.22268686 2.87552292 |
| | 7 | Ge | 1.54170390 1.60460839 1.57038175 |
| | 8 | Ge | 2.30581214 -0.82530186 1.28470289 |
| | 9 | Ge | 1.54170390 1.60460839 -1.57038175 |
| | 10 | Ge | 2.30581214 -0.82530186 -1.28470289 |
| | 11 | Ge | 0.69626404 -2.68828117 0.00000000 |
| | 12 | Ge | 0.58580936 -2.33042790 -2.65527801 |
| | 13 | Ge | 0.58580936 -2.33042790 2.65527801 |
| | 14 | Ge | -0.05419419 0.22268686 -2.87552292 |
| | 15 | Ge | -2.90578517 2.23373215 0.00000000 |
| | 16 | Ge | 1.77292335 3.65470744 0.00000000 |
| | 17 | Mn | -0.11087399 -0.03497387 0.00000000 |
| **$Ge_{16}Mn^{-}$** anion | 1 | Ge | -1.84041300 2.58001800 0.00000000 |
| | 2 | Ge | -2.63470600 -2.47712700 0.00000000 |
| | 3 | Ge | -1.38649000 1.61113800 2.28427100 |
| | 4 | Ge | -1.38649000 1.61113800 -2.28427100 |
| | 5 | Ge | -1.19256900 -0.89156100 -1.36829200 |
| | 6 | Ge | 0.82079800 2.80648900 -1.50440000 |
| | 7 | Ge | 2.69376400 1.85041800 0.00000000 |
| | 8 | Ge | 1.85163700 -3.28196100 0.00000000 |
| | 9 | Ge | 2.09894200 -0.65168400 0.00000000 |
| | 10 | Ge | 0.82079800 2.80648900 1.50440000 |
| | 11 | Ge | 1.18339400 -1.61741900 2.18211800 |
| | 12 | Ge | -0.21029000 4.59558100 0.00000000 |
| | 13 | Ge | -0.44665300 -3.52068100 1.36749800 |
| | 14 | Ge | -0.44665300 -3.52068100 -1.36749800 |
| | 15 | Ge | 1.18339400 -1.61741900 -2.18211800 |
| | 16 | Ge | -1.19256900 -0.89156100 1.36829200 |
| | 17 | Mn | 0.10765400 0.77929000 0.00000000 |

| | | | |
|---|---|---|---|
| **$Ge_{16}Fe$** neutral | 1 | Ge | -1.44171500 -1.27553900 2.07884000 |
| | 2 | Ge | 1.08587500 1.50416300 1.91940700 |
| | 3 | Ge | -1.43956600 1.27213800 2.08118600 |
| | 4 | Ge | 1.08352000 -1.50801900 1.91625200 |
| | 5 | Ge | 2.40570300 -0.00114800 0.00060400 |
| | 6 | Ge | 0.24522300 -2.82754500 -0.00251300 |
| | 7 | Ge | 1.08537300 -1.50477800 -1.91862300 |
| | 8 | Ge | -1.44007100 -1.27051800 -2.08192100 |
| | 9 | Ge | 1.08681000 1.50700100 -1.91596600 |
| | 10 | Ge | -1.43872900 1.27715500 -2.07961500 |
| | 11 | Ge | -2.82135800 0.00132900 -0.00066400 |
| | 12 | Ge | -2.37615900 2.63560300 0.00226400 |
| | 13 | Ge | -2.37920200 -2.63408900 -0.00348900 |
| | 14 | Ge | 0.24853400 2.82746700 0.00256200 |
| | 15 | Ge | 3.10272600 -0.00417500 2.41383400 |
| | 16 | Ge | 3.10469400 0.00064800 -2.41202200 |
| | 17 | Fe | -0.13742700 0.00037700 -0.00016600 |
| **$Ge_{16}Fe^-$** anion | 1 | Ge | 2.65183100 -1.68389000 0.75955300 |
| | 2 | Ge | -1.92022100 -2.33346800 1.08397700 |
| | 3 | Ge | 1.67005400 -2.19246800 -1.57745300 |
| | 4 | Ge | 1.66548800 -0.26132800 2.68451400 |
| | 5 | Ge | -0.72752200 -0.16156000 1.78263300 |
| | 6 | Ge | 2.66717300 1.50093300 1.07032000 |
| | 7 | Ge | 1.67033100 2.46209500 -1.12013600 |
| | 8 | Ge | -3.43330000 1.41712200 -0.66265900 |
| | 9 | Ge | -0.73150700 1.64522000 -0.74671600 |
| | 10 | Ge | 2.69250200 0.18092900 -1.82455600 |
| | 11 | Ge | -1.89525900 0.22989800 -2.55543100 |
| | 12 | Ge | 4.41767800 -0.01038500 0.02480100 |
| | 13 | Ge | -3.43278900 -1.27019300 -0.90812700 |
| | 14 | Ge | -3.44207000 -0.14237100 1.54446000 |
| | 15 | Ge | -1.92002000 2.09782000 1.48567700 |
| | 16 | Ge | -0.72764700 -1.48609700 -1.02694100 |
| | 17 | Fe | 0.97880200 0.00952900 -0.01712700 |

| | | | | | |
|---|---|---|---|---|---|
| **$Ge_{16}Co$** neutral | 1 | Ge | -1.29886700 | 1.39071300 | -2.07456900 |
| | 2 | Ge | 0.90538500 | -1.56791700 | -1.88681500 |
| | 3 | Ge | -1.58358300 | -1.13693600 | -2.03734000 |
| | 4 | Ge | 1.23320400 | 1.40433400 | -1.96587900 |
| | 5 | Ge | 2.39213500 | -0.18205900 | 0.00000200 |
| | 6 | Ge | 0.57532600 | 2.75905300 | -0.00006800 |
| | 7 | Ge | 1.23320500 | 1.40443200 | 1.96581200 |
| | 8 | Ge | -1.29886100 | 1.39080300 | 2.07451000 |
| | 9 | Ge | 0.90538800 | -1.56782200 | 1.88689200 |
| | 10 | Ge | -1.58358300 | -1.13684700 | 2.03738600 |
| | 11 | Ge | -2.78032200 | 0.23972300 | -0.00000200 |
| | 12 | Ge | -2.66933200 | -2.48085500 | 0.00005000 |
| | 13 | Ge | -2.03102800 | 2.86453800 | -0.00006000 |
| | 14 | Ge | -0.05888200 | -2.83043100 | 0.00006900 |
| | 15 | Ge | 3.07434900 | -0.33451500 | -2.40090300 |
| | 16 | Ge | 3.07435200 | -0.33439300 | 2.40091200 |
| | 17 | Co | -0.10534600 | 0.14006300 | 0.00000200 |
| **$Ge_{16}Co^-$** anion | 1 | Ge | 2.62154600 | -1.82771000 | 0.00400100 |
| | 2 | Ge | -2.00658300 | -2.59598400 | 0.00561900 |
| | 3 | Ge | 1.62428100 | -1.35756900 | -2.30261300 |
| | 4 | Ge | 1.62464300 | -1.34717200 | 2.30866600 |
| | 5 | Ge | -0.73147100 | -0.86811800 | 1.43613300 |
| | 6 | Ge | 2.72991500 | 0.88277400 | 1.60042700 |
| | 7 | Ge | 1.69604200 | 2.66357100 | -0.00603400 |
| | 8 | Ge | -3.41367200 | 1.59945800 | -0.00352100 |
| | 9 | Ge | -0.74447100 | 1.79512500 | -0.00380200 |
| | 10 | Ge | 2.72969700 | 0.87535900 | -1.60442700 |
| | 11 | Ge | -1.93793100 | 1.27226100 | -2.24289300 |
| | 12 | Ge | 4.46489000 | -0.05763400 | -0.00001800 |
| | 13 | Ge | -3.43179900 | -0.77319700 | -1.36243700 |
| | 14 | Ge | -3.43185500 | -0.76722100 | 1.36554000 |
| | 15 | Ge | -1.93812100 | 1.28205100 | 2.23748000 |
| | 16 | Ge | -0.73143000 | -0.87445900 | -1.43201600 |
| | 17 | Co | 1.03859900 | 0.11669900 | -0.00012400 |

| | | | |
|---|---|---|---|
| **$Ge_{16}Ni$** neutral | 1 | Ge | 2.14227000 1.07358500 1.08730100 |
| | 2 | Ge | 1.79100800 -2.18846100 -2.20826800 |
| | 3 | Ge | 1.79067500 -0.82133900 2.99794900 |
| | 4 | Ge | 1.79899700 3.00268700 -0.78922000 |
| | 5 | Ge | 2.13730800 -1.48284400 0.38922500 |
| | 6 | Ge | 2.14199600 0.39938800 -1.47372100 |
| | 7 | Ge | -0.21295900 1.84487100 -2.03675800 |
| | 8 | Ge | -0.22096600 -2.05557100 1.82410500 |
| | 9 | Ge | -0.21713200 -0.55078100 -2.69097600 |
| | 10 | Ge | -0.21258900 2.60884100 0.86824700 |
| | 11 | Ge | -0.22051600 -2.68641000 -0.57846600 |
| | 12 | Ge | -0.21702300 0.84247600 2.61453700 |
| | 13 | Ge | -2.13250100 1.65130000 -0.43281000 |
| | 14 | Ge | -2.13777600 -0.44708300 1.64296900 |
| | 15 | Ge | -2.13738300 -1.19645100 -1.21216000 |
| | 16 | Ge | -4.00166900 0.00629400 -0.00249600 |
| | 17 | Ni | -0.10484500 -0.00057400 0.00061700 |
| **$Ge_{16}Ni^-$** anion | 1 | Ge | -2.62662500 -1.53963400 0.89052400 |
| | 2 | Ge | 2.22133500 -2.23598300 1.34607200 |
| | 3 | Ge | -1.46004300 0.00017200 2.52055300 |
| | 4 | Ge | -1.71506200 -2.32270700 -1.30595900 |
| | 5 | Ge | 0.69266100 -1.77221500 -0.60962500 |
| | 6 | Ge | -2.85353800 -0.00017100 -1.57469500 |
| | 7 | Ge | -1.71508600 2.32246300 -1.30624800 |
| | 8 | Ge | 3.28523300 1.36753800 -1.07502200 |
| | 9 | Ge | 0.69313400 1.77164600 -0.61088400 |
| | 10 | Ge | -2.62637100 1.53985500 0.89033300 |
| | 11 | Ge | 2.22163700 2.23686800 1.34467200 |
| | 12 | Ge | -4.59063900 0.00033500 0.31868900 |
| | 13 | Ge | 3.62476300 0.00023700 1.24157800 |
| | 14 | Ge | 3.28485400 -1.36839600 -1.07429700 |
| | 15 | Ge | 1.36794400 -0.00083500 -2.42121700 |
| | 16 | Ge | 0.89102500 0.00076400 1.61375500 |
| | 17 | Ni | -0.79454200 0.00007500 -0.21511800 |

| | | | |
|---|---|---|---|
| **$Ge_{16}Cu$** neutral | 1 | Ge | 2.70326600 1.50864000 1.09806300 |
| | 2 | Ge | -1.95653200 2.11248600 1.53555700 |
| | 3 | Ge | 1.63175300 -0.27989500 2.64057900 |
| | 4 | Ge | 1.63133300 2.42694900 -1.07800400 |
| | 5 | Ge | -0.75682000 1.57753200 -0.70168200 |
| | 6 | Ge | 2.70279400 0.19701500 -1.85460200 |
| | 7 | Ge | 1.63197400 -2.14718100 -1.56277100 |
| | 8 | Ge | -3.40581000 -1.28930900 -0.93722300 |
| | 9 | Ge | -0.75645000 -1.39703600 -1.01447300 |
| | 10 | Ge | 2.70356400 -1.70510800 0.75709300 |
| | 11 | Ge | -1.95613400 -2.38595100 1.06245100 |
| | 12 | Ge | 4.40027300 0.00032300 -0.00019100 |
| | 13 | Ge | -3.40725100 -0.16650600 1.58366100 |
| | 14 | Ge | -3.40623100 1.45598100 -0.64925100 |
| | 15 | Ge | -1.95452300 0.27288900 -2.59714200 |
| | 16 | Ge | -0.75764700 -0.18068100 1.71765200 |
| | 17 | Cu | 1.05097100 -0.00016500 0.00031200 |
| **$Ge_{16}Cu^-$** anion | 1 | Ge | 2.75962100 -1.75020800 0.04241100 |
| | 2 | Ge | -2.02141200 -2.58808200 0.06238300 |
| | 3 | Ge | 1.67309000 -1.37988100 -2.25223700 |
| | 4 | Ge | 1.67299900 -1.26866000 2.31628300 |
| | 5 | Ge | -0.78927900 -0.83291400 1.50269800 |
| | 6 | Ge | 2.76336500 0.90784900 1.50214300 |
| | 7 | Ge | 1.68653400 2.63658000 -0.06433000 |
| | 8 | Ge | -3.46635300 1.58905200 -0.03824900 |
| | 9 | Ge | -0.78552700 1.73453400 -0.04107400 |
| | 10 | Ge | 2.76408800 0.83346000 -1.54412300 |
| | 11 | Ge | -2.00358800 1.23685600 -2.26834400 |
| | 12 | Ge | 4.62598100 -0.00327400 0.00041900 |
| | 13 | Ge | -3.47171000 -0.81699800 -1.35245900 |
| | 14 | Ge | -3.47231500 -0.75094700 1.38838100 |
| | 15 | Ge | -2.00416100 1.34342000 2.20756500 |
| | 16 | Ge | -0.78890000 -0.90388300 -1.46094100 |
| | 17 | Cu | 0.94628100 0.01445000 -0.00058100 |

| | | | |
|---|---|---|---|
| **$Ge_{16}Zn$** neutral | 1 | Ge | 2.82325100 -1.76769300 0.18133000 |
| | 2 | Ge | -2.04498000 -2.59711800 0.26549100 |
| | 3 | Ge | 1.69692100 -1.54840100 -2.13573700 |
| | 4 | Ge | 1.69686000 -1.08220600 2.40510200 |
| | 5 | Ge | -0.84607700 -0.72479200 1.59216400 |
| | 6 | Ge | 2.82294900 1.03928900 1.44598700 |
| | 7 | Ge | 1.70549100 2.62176900 -0.26922600 |
| | 8 | Ge | -3.49774600 1.58426500 -0.16225400 |
| | 9 | Ge | -0.84282700 1.75141900 -0.17746000 |
| | 10 | Ge | 2.82310500 0.72299100 -1.62616400 |
| | 11 | Ge | -2.02666900 1.06540000 -2.37929400 |
| | 12 | Ge | 4.67044800 -0.00046700 0.00059500 |
| | 13 | Ge | -3.50261600 -0.92138600 -1.28884700 |
| | 14 | Ge | -3.50442600 -0.64236500 1.44527600 |
| | 15 | Ge | -2.02985800 1.52456100 2.11541400 |
| | 16 | Ge | -0.84490100 -1.03234600 -1.41152600 |
| | 17 | Zn | 0.96114400 0.00755000 -0.00090800 |
| **$Ge_{16}Zn^-$** anion | 1 | Ge | 2.90746800 0.71336000 -1.60505000 |
| | 2 | Ge | -2.07794600 1.04216600 -2.34153400 |
| | 3 | Ge | 1.70487800 2.59030300 -0.27291000 |
| | 4 | Ge | 1.70431300 -1.53253500 -2.10626300 |
| | 5 | Ge | -0.84241100 -1.01700000 -1.39786300 |
| | 6 | Ge | 2.90790100 -1.74609700 0.18456600 |
| | 7 | Ge | 1.70452500 -1.05802900 2.38033000 |
| | 8 | Ge | -3.60257500 -0.63083700 1.41695100 |
| | 9 | Ge | -0.84124500 -0.70277600 1.57833500 |
| | 10 | Ge | 2.90811600 1.03275300 1.41983500 |
| | 11 | Ge | -2.07740600 1.50646200 2.07348200 |
| | 12 | Ge | 4.78943700 -0.00005300 -0.00072700 |
| | 13 | Ge | -3.60316200 1.54232500 -0.16211800 |
| | 14 | Ge | -3.60363900 -0.91082600 -1.25455800 |
| | 15 | Ge | -2.07811200 -2.54895300 0.26873000 |
| | 16 | Ge | -0.84206600 1.71968200 -0.18096100 |
| | 17 | Zn | 1.00471700 0.00005800 -0.00026200 |
